# The Association between CFTR Gene Mutation Heterozygosity and Asthma Development: A Systematic Review

**DOI:** 10.3390/jcm12062403

**Published:** 2023-03-21

**Authors:** Despoina Koumpagioti, Dafni Moriki, Barbara Boutopoulou, Vasiliki Matziou, Ioanna Loukou, Kostas N. Priftis, Konstantinos Douros

**Affiliations:** 1 Department of Nursing, National and Kapodistrian University of Athens, 11527 Athens, Greece; 2 Third Department of Pediatrics, School of Medicine, Attikon University Hospital, National and Kapodistrian University of Athens, 12462 Athens, Greece; 3 Department of Cystic Fibrosis, Aghia Sophia Children’s Hospital, 11527 Athens, Greece

**Keywords:** cystic fibrosis, cystic fibrosis transmembrane conductance regulator, heterozygote, asthma

## Abstract

Asthma is caused by complex interactions between environmental and genetic factors. Various genes have been implicated as potential risk factors in the development of asthma; among them is cystic fibrosis transmembrane conductance regulator (CFTR) gene. The aim of this systematic review was to investigate the association of CFTR mutation heterozygosity with the development of asthma, by updating the existing data with recent studies’ findings. Therefore, a systematic review of the literature was conducted on Pubmed, ESBCO (Cinahl) and Scopus Databases up to December 2022. After the eligibility assessment, 17 studies were included in this review. Nine of them supported a lack of relationship between CFTR mutation heterozygosity and asthma susceptibility, and eight reported a positive association. Consequently, more extensive research is needed through high-quality studies to provide valid evidence and highlight the clinical benefits of identifying CFTR mutations in asthma patients, their impact on asthma severity, or treatment perspectives.

## 1. Introduction

Asthma is a heterogeneous disease, characterized by chronic airway inflammation, caused by complex interactions between genetic and environmental factors [1]. Various genes have been implicated in the development of asthma; among them is cystic fibrosis transmembrane conductance regulator (CFTR) gene [2]. CFTR regulates chloride and bicarbonate transport across epithelial surface of multiple organs, including the lungs. Changes in ion transport have been related to asthma pathogenesis [3].

Mutations or polymorphisms in CFTR gene result in cystic fibrosis (CF), an autosomal recessive disease [4]. More than 2000 CFTR variants have been identified in CF patients and CF-related conditions [5]. Heterozygosity for CFTR mutations (known as CF carriers) is characterized by the absence of CF symptoms, although it has been linked with an elevated risk of CF-related conditions such as male infertility; chronic pancreatitis; chronic sinusitis; bronchiectasis; nontuberculous mycobacterial infections, and asthma [6].

CFTR gene and its role in asthma development has been studied extensively over the past few years. Several studies reported an increased asthma risk in CF carriers compared to non-carriers [7,8,9,10]. Carriers for CFTR mutations have been estimated as up to 1.9% of asthma in Asians and 1.6% of asthma in Europeans, indicating the significant CF heterozygosity’s contribution to asthma cases among Asian and European populations, according to meta-analysis findings [11]. Conversely, other studies showed no significant relationship between CFTR mutation heterozygosity and asthma susceptibility [12,13,14,15]. The aim of this systematic review was to investigate the association of CFTR mutation heterozygosity with the development of asthma, by updating the existing data with recent studies’ findings.

## 2. Materials and Methods

The present systematic review was performed following the Preferred Reporting items for systematic reviews and meta-analyses (PRISMA) guidelines [16].

### 2.1. Literature Search

The literature search was conducted on PubMed, ESBCO (Cinahl) and Scopus databases from the inception to December 2022, using the MESH terms “cystic fibrosis”, “cystic fibrosis transmembrane conductance regulator”, “heterozygote”, and “asthma”. Studies were independently screened by two reviewers, according to the above inclusion criteria. Any discrepancies were solved by consensus.

### 2.2. Eligibility Criteria and Study Selection

In this systematic review, we included studies that were published from 1 January 1998 until 31 December 2022, in the English language, and studies that investigated and provided distinguished results concerning the association between CFTR mutation heterozygosity and the development of asthma.

### 2.3. Data Extraction

Two reviewers independently extracted data from the selected studies. The extracted data included study details (author’s name, year of publication, country, and study design); participants’ characteristics (age, number, and comparison groups); asthma diagnosis; the examined CFTR mutations; asthma susceptibility, and the association of CFTR mutation heterozygosity with asthma development.

## 3. Results

### 3.1. Study Selection

**Figure 1:**
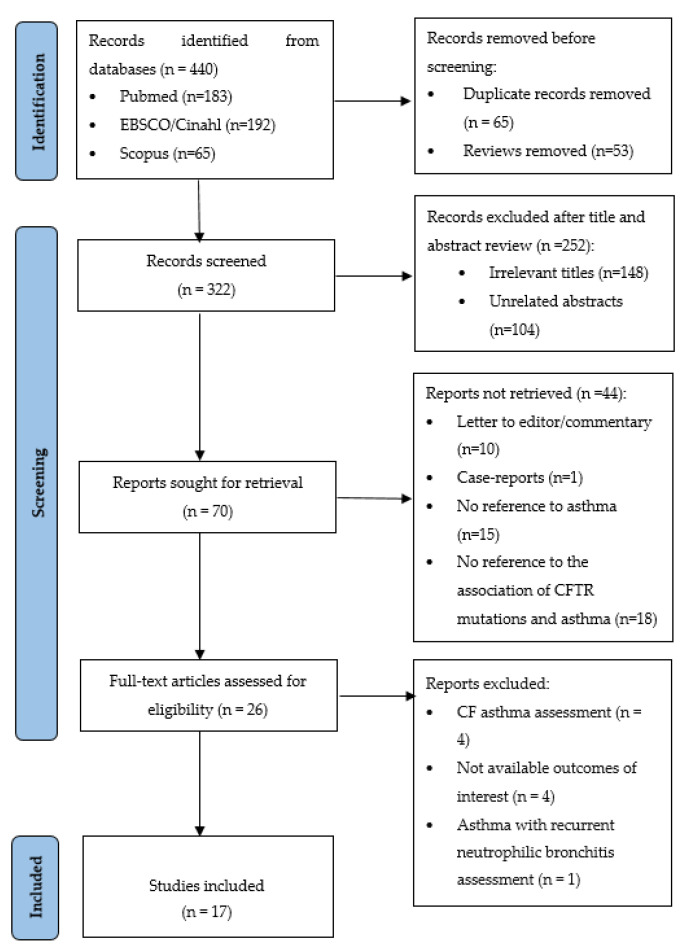
PRISMA diagram for study selection process.

A total of 440 potentially eligible studies were identified for screening, during the database search. After removing duplicates and reviews (*n* = 118), 322 studies remained for further examination. Of these, 252 studies were excluded manually after title and abstract review. The full text of 26 articles was assessed for eligibility. Finally, 17 studies met the inclusion criteria and were included in this systematic review. Figure 1 depicts the study selection process.

### 3.2. Studies’ Characteristics

A total of 17 studies that examined the association of CFTR mutation heterozygosity with asthma development were included in this systematic review. Three studies were conducted in India [10,17,18], two in Greece [19,20], two in Denmark [7,21], two in the USA [2,6], one in Italy [12], one in Spain [8], one in France [13], one in Singapore [9], one in China [22], one in Korea [15], one in Norway [14], and one study was multinational [23].

The majority of the studies (*n* = 13) were case-control [8,9,10,12,13,14,15,17,18,19,20,22,23], two cross-sectional [7,21], and two retrospective cohort studies [2,6].

The sample sizes ranged from 54 to 99,010 participants, with the age range being 9 months to 93 years. Eight out of 17 studies enrolled adults as participants [7,8,9,12,20,21,22,23], five studies children exclusively [2,14,15,17,18], three both adults and children [6,13,19], and one study did not report the participants’ age [10].

According to asthma diagnosis, in eight studies asthma was diagnosed by a physician [2,8,9,10,14,15,20,22], in four studies through asthma questionnaires [7,12,23] or self-report [13], in two studies asthma cases should follow the criteria of receiving asthma medication; hospitalization; current wheezing; and presenting with a first episode of wheeze along with a positive family history of asthma [17,18], and in three studies there was no report about the way asthma was diagnosed [6,19,21].

The characteristics of the included studies are shown in Table 1.

### 3.3. CFTR Mutation Heterozygosity and the Risk of Asthma

The results of the 17 selected studies regarding the association between CFTR mutation heterozygosity and asthma development reported no association according to the majority of the studies [12,13,14,15,17,20,21,22,23], while eight out of 17 studies noted a positive association [2,6,7,8,9,10,18,19].

Lowenfels et al., in their multinational study, found no difference with regard to asthma prevalence between F508del carriers and non-carriers, with the estimated asthma prevalence in F508del heterozygotes being 9.6%, similar to that reported by Dahl et al., although the odds ratio was only slightly raised [23]. In the French EGEA study, no significant association was noted between asthma and heterozygosity for F508del (odds ratio (OR) =1.13, 95% confidence interval (CI): 0.36–3.52, *p* = 0.83); R75Q (OR = 0.46, 95% CI: 0.16 (OR = 0.79, 95% CI: 0.30–2.07, *p* = 0.62); L997F (OR = 0.68, 95% CI: 0.34–1.37, *p* = 1.0); M470V (*p* = 0.66), and IVS8-(T) n, 5T/– (OR = 0.51, 95% CI: 0.27–0.99, *p* = 0.05) mutations [13]. Castellani et al., identified no significant difference between CFTR heterozygosity and asthma (*p* > 0.05), with 4.59% of asthma cases and 3.98% of controls being heterozygotes [12]. In another case-control study, no association was found between asthma and CFTR heterozygosity (*p* = 0.43), as well [14].

Douros et al. observed that 4.2% of carriers for F508del and 7.6% of non-carriers were characterized as asthmatics, concluding that there was an absence of significant difference in the prevalence of asthma between carriers and non-carriers (OR = 0.61, 95% CI: 0.23–1.61, *p* = 0.32) [20]. Similarly, Kim et al. documented the lack of significant association between CFTR heterozygosity and asthma (*p* > 0.05) [15]. Wang et al. examined the role of CFTR variations, poly-T, TG-repeats, and M470V in susceptibility to asthma or chronic bronchitis compared to healthy controls and found that the frequency of the T5/T7 heterozygote was virtually identical for all groups [22]. Dixit et al. showed that among 24 CFTR mutations, the heterozygous allele was found only in R553X mutation in 1.6% (*n* = 4) among asthma cases and 0.4% (*n* = 2) among controls, while no significant difference in the genotype and allele frequency of R553X mutation (OR = 1.339, 95% CI: 0.755–2.374, *p* = 0.685) was reported [17].

Additionally, a Copenhagen general population study that examined if F508del carriers had increased morbidity and mortality versus non-carriers in the general population, showed that there was a nominal difference in asthma between carriers and non-carriers (7.17% versus 6.8%), but it did not reach a statistical difference, yielding an unadjusted OR of 1.05 (95% CI: 0.91–1.21) [21].

Conversely, eight studies reported a positive association between CFTR mutation heterozygosity and asthma development [2,6,7,8,9,10,18,19].

In a cross-sectional study, the prevalence of asthma in CFTR heterozygotes was significantly higher (9%) than in non-carriers (6%), (*p* = 0.04). Moreover, the odds ratio for asthma and daily intake of asthma medication among heterozygotes for F508del mutation were 2.0 (95% CI: 1.2–3.5, *p* = 0.02) and 2.0 (95% CI: 1.1–3.4, *p* = 0.03), respectively [7]. A Spanish case-control study, based on 144 asthma cases and 41 controls from the general population (spouses of CF carriers) (control group 1), showed an overrepresentation of heterozygotes for R75Q, G576A, R668C, and L997F amino acids variants in the asthma cases, with L997F (2.1% in asthma patients) being the most frequent whilst, in the second control group of 184 anonymous blood donors, the proportion of the above variants was similar to the one of asthma cases (R75Q (1.6% general population individuals versus 2.8% asthma patients); G576A (2.7% general population individuals versus 2.1% asthma patients) and R668C (4.3% general population individuals versus 3.5% asthma patients)). These missense mutations were presented together with a high frequency of the hyper-functional allele M470 (asthma cases with missense mutations (52%) than asthmatic cases without mutations (38%) (*p* = 0.08) and the general population (33%) (*p* = 0.04)) and an absence of the 5T allele, putatively contributing to the genetic variability of asthma [8]. In a study by Tzetis et al., a statistically significant increase in CFTR mutations (45% heterozygotes, *p* < 0.05) and of the IVS8–5T allele (10% carriers, *p* < 0.05), in asthma, was found [19]. Ngiam et al. recruited 14 participants with severe asthma, 40 unaffected controls and 96 unselected population samples, examining four CFTR mutations. A higher incidence of the Q1352H heterozygosity was observed in the severe asthma group (14.3%), compared to normal controls (2.5%), and an estimated population heterozygote frequency of 4.2%. Similarly, I556V and 12TG5T polymorphisms heterozygosity were overrepresented in the severe asthma patients (21.4% and 14.3%, respectively) than normal controls (10% and 2.5%, respectively) and an estimated population frequency of 12.5% and 9.7%, respectively. However, no statistical significance was found (*p* = 0.172; *p* = 0.415; and *p* = 0.640 respectively) [9].

In the study of Awasthi et al., 25 carriers for five CFTR mutations (F508del, G542X, G551D, R117H, and W1282X) were identified among 200 asthmatic children, with the most prevalent mutation the G551D (*n* = 12). Carriers for G551D mutation had a significantly increased risk for persistent asthma (OR = 6.6, 95% CI: 1.41–31.18, *p* = 0.006) [18]. A case-control study of 250 asthma cases and 400 controls, reported an excessive frequency (%) of heterozygous individuals among asthma cases than in the general population (24% and 9.3%, respectively, *p* < 0.0001) [10]. Miller et al. in a population-based retrospective matched-cohort study, evaluated if CF carriers were more susceptible to 59 examined CF-related conditions (asthma included), and found that CFTR mutation heterozygosity was significantly associated with an increased risk of asthma (OR =1.36, 95% CI: 1.29–1.43, *p* < 0.001) [6]. Thilakaratne et al., in a retrospective cohort study which was conducted in a pediatric population, revealed that CF carriers had a higher risk of asthma compared to population controls (adjusted risk ratio (aRR) = 1.29, 95% CI: 0.98–1.69, *p* < 0.1), whilst genotypes with the greatest asthma risk were F508del with an intron 10 T7 or (TG) 11T5 in trans (aRR = 1.52, 95% CI: 1.10–2.12) [2].

## 4. Discussion

In this systematic review, we examined the association of CFTR mutation heterozygosity with asthma development. Our review showed conflicting results, and essentially a dichotomy in the literature given that nine studies supported no association [12,13,14,15,17,20,21,22,23], while eight out of 17 studies reported a positive association between CFTR heterozygosity and the development of asthma [2,6,7,8,9,10,18,19]. However, there was a lack of crucial information by some studies regarding the way asthma was diagnosed [6,19,21], the age of participants [10], or the examined CFTR mutations [6] highlighting their methodological bias. Taking into consideration that three of these studies presented a positive association [6,10,19], the findings have to be interpreted with caution.

On the contrary, a meta-analysis of 15 studies that was held to determine the risk of asthma in CF heterozygotes found that the asthma risk in carriers was significantly higher than in non-carriers (OR 1.61, 1.18–2.21). Moreover, the analysis of high-quality studies in which asthma was physician-diagnosed, patients were older than 18 years, or the study size was up to 500 participants, showed that the summary ORs remained significantly increased at 1.39 to 1.96, supporting the hypothesis that CF heterozygosity is a risk factor for asthma [11].

CFTR mutation heterozygosity might influence the clinical expression of asthma. Awasthi et al. found that the symptoms of asthma, namely, wheezing along with shortness of breath, cough, and disturbed sleep, were more severe among carriers than non-carriers, while carriers for G551D mutation had an increased risk for persistent asthma [18]. Additionally, Ngiam et al. showed that heterozygotes for Q1352H, I556V and 12TG5T mutations were overrepresented in the severe asthma group than in normal controls [9].

CFTR heterozygosity has a controversial impact on the establishment of a detectable silent obstructive pulmonary profile. Four studies assessed that the percent predicted Forced Expiratory Volume in one second (FEV_1_) and Forced Vital Capacity (FVC) were lower among carriers as compared to non-carriers [7,17,18,20]. Contrariwise, Lowenfels et al. showed that F508del carriers and non-carriers did not differ with regard to FEV_1_ and bronchial responsiveness (BHR) [23]. Munthe-Kaas et al. found a lack of associations between CFTR mutations (IVS8(TG)mTn haplotypes) and lung function, BHR and increased nitrogen oxide (FeNO); the percentage of CF heterozygosity was higher in subjects without reduced lung function, BHR or increased FeNO levels than in those with the clinical traits. Indeed, IVS8(TG)11T7 haplotype was found to be possibly protective against reduced lung function, posing the question if these polymorphisms are unrelated to asthma but they can influence lung physiology in the general population [14]. In addition, CFTR heterozygosity has been linked with recurrent airway infections in severe asthma. Priel et al. conducted a retrospective chart review in order to characterize the clinical features of CF carriers with asthma and recurrent neutrophilic inflammation and showed that CF carriers are overrepresented in their study. This finding supports the role of CFTR hypofunction in the predisposition of some patients with asthma to recurrent respiratory infections [24].

Genetic variants of CFTR gene may be possibly associated with certain asthma phenotypes. Crespo-Lessman et al. showed that asthma phenotype with mucus hypersecretion may be related with an intronic polymorphism in the CFTR gene (NM_000492.3:c.1680-870T>A), and these patients may have a poorer clinical outcome characterized by severe disease and poorer asthma control with a non-allergic inflammatory phenotype [25]. Additionally, CFTR mutations have probably been linked to asthma severity. Riolo et al. compared the clinical characteristics, including features of CFTR-related disorders and the prevalence of CFTR mutations in individuals with severe asthma and non-severe asthma, concluding that features of CFTR-related disorders (rhinosinusitis, nasal polyposis, and bronchiectasis) were more prevalent in severe asthma than non-severe asthma, while 11% of severe asthma patients had CFTR mutations, suggesting a possible association between CFTR mutations and asthma severity [26].

As it was expected, F508del was one of the most frequently studied or found mutations among studies [2,7,8,10,12,13,14,18,20,23]. Some studies reported a positive association of F508del heterozygosity with an increased vulnerability to asthma [2,7,10], while others mentioned no association [13,18,20,23]. Nevertheless, Schroeder et al. evidenced that F508del CF allele had a protective role against asthma in childhood and early adult life [27]. Another common mutation is G551D for which two case-control studies found it to be associated with asthma, with a higher prevalence in asthma groups as compared to controls. As for the G542X mutation, no difference in its frequency was revealed among asthma cases and controls [10,18]. Concerning the M470V polymorphism, it was found at a higher but not significant frequency, in asthmatic patients with missense mutations (52%) than in asthmatic patients without mutations (38%) (*p* = 0.08) and the general population (33%), according to Lazaro et al. [8]. Wahabi et al. observed that there were no significant differences in the M470V allelic distribution in asthma cases and healthy controls, although they found a significant difference in the genotype frequencies between the two groups, as the *v*/*v* (positive control) genotype was overrepresented in the asthmatic group, whereas the M/V (heterozygous control) genotype was more frequent in the healthy subjects [28]. Regarding the ethnic background, the majority of the selected studies were conducted in Europe (*n* = 8), where five of them revealed no association between CFTR heterozygosity and asthma [12,13,14,20,21] and three found a positive association [7,8,19]. Six studies were conducted in Asia, with three of them reporting a positive association [15,17,22] and the rest showing no association [9,10,18]. Nielsen et al., in their meta-analysis, estimated that in Asian countries the risk for developing asthma in CF heterozygotes was remarkably higher than among studies conducted in European countries (ORs: 2.78 vs. 1.23), suggesting that environmental and sociodemographic factors related to Asian areas might influence the association between CFTR heterozygosity and asthma [11]. Moreover, two studies were conducted in North America, and both observed a positive association [2,6]. Miller et al. assessed the association of CFTR heterozygosity with the risk of CF-related conditions, including asthma, and showed that CF carriers were at increased risk for most conditions associated with CF. The authors noted that since more than 10 million in the US are CF carriers, the disease burden attributable to the CF carrier state is likely substantial [6].

A strength of the present study is the systematic literature search of recently published studies regarding the association of CFTR heterozygosity and asthma development, which provides updated feedback on this particular topic. However, a primary limitation of our systematic review is that there was heterogeneity among studies regarding the sample sizes; age groups; the way asthma was diagnosed, and the adjusted confounders, along with the methodological bias of some studies, the overwhelming numerical superiority of controls compared to cases and the genotyping for only one CFTR mutation by some studies.

Our review revealed an apparent contradiction among studies’ results regarding the relationship between CFTR mutation heterozygosity and asthma risk. Possible reasons, indicated by other authors as well, that justify this disagreement, include the difference in ethnic groups, or the influence of environmental factors on asthma pathogenesis besides the genetic predisposition [29].

## 5. Conclusions

We cannot draw a definite conclusion on the association between CFTR heterozygosity and asthma development. In any case, the fact that several studies supported that CF carriers were at an increased risk of developing asthma cannot be disregarded. Therefore, more extensive research, through high-quality studies, is needed in order to validate evidence in this field and to highlight the clinical benefits of identifying CFTR mutations in asthma patients, their impact on asthma severity, or treatment perspectives.

## Figures and Tables

**Table 1:** Characteristics of the included studies.

Author	Country	Study Design	Sample Characteristics	CFTR Gene Mutations	Definition of Asthma	Asthma Susceptibility	Results
Lowenfels et al. [23]	Multinational	Case-control	111 carriers (mean age (years): 53.4), 688 controls (mean age: 54.6)	ΔF508	Questionnaire	−	No difference with regard to asthma prevalence between F508del carriers and non-carriers, estimated at 9.6%, similar to that reported by Dahl et al. [9], but the odds ratio was only slightly raised.
Dahl et al. [7]	Denmark	Cross-sectional	250 carriers (mean (range) age (years): 59 (23–86)), 8891 non-carriers (mean (range) age (years): 58 (21–93))	ΔF508	Questionnaire	+	Prevalence of asthma in carriers: significantly higher (9%) than in non-carriers (6%), (*p* = 0.04). OR for asthma and daily intake of asthma medication among carriers 2.0 (95% CI: 1.2–3.5, *p* = 0.02) and 2.0 (95% CI: 1.1–3.4, *p* = 0.03) respectively.
Lazaro et al. [8]	Spain	Case-control	144 asthma cases (CFTR^+^ (mean age: 57.2 years), (CFTR^-^ (mean age: 62.8 years), 41 controls for general population (control group 1) 184 controls form anonymous blood donors (control group 2)	Complete CFTR screening by multiplex denaturing gradient gel electrophoresis and for 15 exons and for 12 exons single-strand conformation analysis (∆F508, G542X, IVS8-6 (5T), R75Q, G576A, R668C, L997F)	Physician-diagnosed	+	Overrepresentation of heterozygotes for R75Q, G576A, R668C, and L997F amino acids variants in asthma cases, with L997F (2.1% in asthma patients) being the most frequent, whilst in the control group 2 no difference in the variants proportions was found. High frequency of M470 allele in asthma cases with missense mutations (52%) than in asthmatic cases without mutations (38%) (*p* = 0.08) and general population (33%) (*p* = 0.04) and an absence of the 5T allele.
de Cid et al. [13]	France	Case-control	247 asthma cases (mean age (yeas) ± SD (30.2 ± 17.9), 174 controls (mean age ± SD (34.7 ± 16.1)	ΔF508, R75Q, G576A, R668C, L997F, M470V, IVS8-(T) n, 5T/–	Self-report	−	No significant association between asthma and heterozygosity for ΔF508 (*p* = 0.83), R75Q (*p* = 0.14), G576A (*p* = 0.84), R668C (*p* = 0.62), L997F (*p* = 1.0), M470V (*p* = 0.66), IVS8-(T) n, 5T/– (*p* = 0.05).
Tzetis et al. [19]	Greece	Case-control	20 asthma cases, 52 controls Age range: 9 months-16 year-old and 25–67 year-old	27 exons and neighboring intronic regions of CFTR, cryptic splice mutation 3849 + 10KbC > T, IVS8-polyT	Not mentioned	+	Significant increase of CFTR gene mutations (45% heterozygotes, *p* < 0.05) and of IVS8-5T allele (10% carriers, *p* < 0.05) in asthma cases.
Castellani et al. [12]	Italy	Case-control	261 carriers (mean age (years): 44), 201 controls (mean age (years): 36)	15 CFTR gene mutations	Questionnaire	−	No significant difference between CF heterozygosity and asthma (*p* > 0.05), with 4.59% of asthma cases and 3.98% of controls to be heterozygotes.
Ngiam et al. [9]	Singapore	Case-control	14 cases with severe asthma, 40 controls (median age (years): 51.5)	1125T, 1556V, Q1352H, intron8 12TG5T	Physician-diagnosed	+	Higher incidence of the Q1352H, I556V, 12TG5T polymorphism heterozygosity in the severe asthma group (14.3%, 21.4% and 14.3% respectively), versus normal controls (2.5%, 10% and 2.5% respectively) and an estimated population heterozygote frequency of 4.2%, 12.5% and 9.7% respectively. No statistical significance, was found (*p* = 0.172; *p* = 0.415; and *p* = 0.640 respectively).
Munthe-Kaas et al. [14]	Norway	Case-control	236 children with asthma, 461 controls (mean age: 10.4)	ΔF508, R117H, 4005 + 2T → C, 394delTT, IVS8 Tn(TG)m	Physician-diagnosed	−	No association between CF heterozygosity and asthma (*p* = 0.43).
Douros et al. [20]	Greece	Case-control	214 carriers (mean age: 36.32), 185 non-carriers (mean age: 32.32)	ΔF508	Physician-diagnosed	−	No significant difference in the prevalence of asthma between carriers and non-carriers (*p* = 0.32).
Kim et al. [15]	Korea	Case-control	48 children with asthma (mean age (years) ± SD: 9.48 ± 2.04), 48 controls (mean age (years) ± SD: 9.63 ± 2.44)	14 CFTR gene mutations	Physician-diagnosed	−	No association between CF heterozygosity and asthma (*p* > 0.05).
Wang et al. [22]	China	Case-control	72 asthma cases (age range: 19–72 year-old), 117 controls (age range: 18–68 year-old)	Poly-T, TG-repeats, M470V polymorphisms	Physician-diagnosed	−	No difference in the frequency of T5/T7 heterozygote among asthma cases and controls.
Awasthi et al. [18]	India	Case-control	200 children with asthma (mean age (months) ± SD: 77.22 ± 42.66), 180 controls (mean age (months) ± SD: 78.94 ± 43.01)	ΔF508, G542X, G551D, R117H, W1282X	Current presence of wheezing, receiving asthma medication, hospitalization, first wheezing episode with positive family history of asthma	+	Significantly increased risk for persistent asthma for carriers for G551D (*p* = 0.006). Symptoms of asthma presenting wheeze along with shortness of breath, cough, and disturbed sleep higher among carriers than non-carriers (*p* = 0.037). % predicted FEV_1_, FVC significantly lower among carriers than non-carriers (*p* = 0.014 and *p* = 0.028 respectively).
Muthuswamy et al. [10]	India	Case-control	250 asthma cases, 400 controls	ΔF508, G524X, G551, R117H, S549N, IVS8-5T	Physician-diagnosed	+	Significantly higher frequency (%) of heterozygous individuals among asthma cases than in general population (24% and 9.3% respectively, *p* < 0.0001).
Dixit et al. [17]	India	Case-control	250 asthma cases (mean age (months) ± SD: 78.22 ± 43.28) 250 controls (mean age (months) ± SD: 77.12 ± 41.02)	24 CFTR mutations	Current presence of wheezing, receiving asthma medication, hospitalization, first wheezing episode with positive family history of asthma	−	No significant difference in the genotype and allele frequency of R553X mutation (*p* = 0.685) among heterozygotes and controls.
Miller et al. [6]	USA	Population-based retrospective matched-cohort	19,802 carriers (11,312 asthma cases), 99,010 controls (8637 asthma cases) (age at first enrollment: 0 to ≥47 year-old)	Not mentioned	Not mentioned	+	Significant association between CFTR gene mutations and an increased risk of asthma *p* < 0.001).
Çolak et al. [21]	Denmark	Cross-sectional	105,176 non-carriers (median age (years): 58.2), 2858 carriers (median age: 59.3). Of these: 7430 asthma cases, 100,604 controls	Phe508del	Not mentioned	−	Nominal difference in asthma between carriers and non-carriers (7.17% versus 6.8%)-no statistical difference, (unadjusted OR of 1.05 (95% CI: 0.91–1.21).
Thilakaratne et al. [2]	USA	Retrospective cohort	941 carriers (mean age (range): 8.8 (years) (5–12)), 4805 controls, (mean age (range): 8.7 (5–12))	Intron 9 (TG)mTn Poly-Variant Tract, the highest risk was found in subjects who had an F508del variant on one allele and a (TG)11T5 or T7 on the second allele	Physician-diagnosed	+	CF carriers had higher risk of asthma compared to population controls (*p* < 0.1) *.

CF: cystic fibrosis, FEV_1_: Forced Expiratory Volume in 1 s, FVC: Forced Vital Capacity, CFTR: cystic fibrosis transmembrane conductance regulator, OR: odds ratio, CI: confidence interval, SD: standard deviation. * Estimates that approach significance with *p* < 0.1 [2]. CFTR+ and CFTR– refer to patients with or without CFTR missense mutations, respectively [8].

## Data Availability

Not applicable.
